# Non-thyroidal illness syndrome and SARS-CoV-2-associated multisystem inflammatory syndrome in children

**DOI:** 10.1007/s40618-021-01647-9

**Published:** 2021-07-26

**Authors:** V. Calcaterra, G. Biganzoli, D. Dilillo, S. Mannarino, L. Fiori, G. Pelizzo, E. Zoia, V. Fabiano, P. Carlucci, A. Camporesi, C. Corti, G. Mercurio, F. Izzo, E. Biganzoli, G. Zuccotti

**Affiliations:** 1 Pediatric and Adolescent Unit, Department of Internal Medicine, University of Pavia, Via Aselli 2, 27100 Pavia, Italy; 2 Pediatric Department, “V. Buzzi” Children’s Hospital, 20154 Milan, Italy; 3 Pharmacogenomics and Precision Therapeutics Master Degree, University of Milan, 20142 Milan, Italy; 4 Pediatric Cardiology Unit, “Vittore Buzzi” Children’s Hospital, 20154 Milano, Italy; 5 Pediatric Surgery Department, “Vittore Buzzi” Children’s Hospital, University of Milan, 20154 Milan, Italy; 6 Anesthesia and Intensive Care Unit, “Vittore Buzzi” Children’s Hospital, 20154 Milano, Italy; 7 Department of Biomedical and Clinical Science “L. Sacco”, University of Milan, 20157 Milan, Italy; 8 Department of Clinical Sciences and Community Health and DSRC, University of Milan, 20122 Milan, Italy

**Keywords:** Children, Non-thyroidal illness syndrome, Multisystem inflammatory syndrome, SARS-CoV-2, Overall Severity Score

## Abstract

**Purpose:**

COVID-19 disease may result in a severe multisystem inflammatory syndrome in children (MIS-C), which in turn may alter thyroid function (TF). We assessed TF in MIS-C, evaluating its impact on disease severity.

**Methods:**

We retrospectively considered children admitted with MIS-C to a single pediatric hospital in Milan (November 2019–January 2021). Non-thyroidal illness syndrome (NTIS) was defined as any abnormality in TF tests (FT3, FT4, TSH) in the presence of critical illness and absence of a pre-existing hormonal abnormality. We devised a disease severity score by combining severity scores for each organ involved. Glucose and lipid profiles were also considered. A principal component analysis (PCA) was performed, to characterize the mutual association patterns between TF and disease severity.

**Results:**

Of 26 (19 M/7F) patients, median age 10.7 (IQR 5.8–13.3) years, 23 (88.4%) presented with NTIS. A low FT3 level was noted in 15/23 (65.3%), while the other subjects had varying combinations of hormone abnormalities (8/23, 34.7%). Mutually correlated variables related to organ damage and inflammation were represented in the first dimension (PC1) of the PCA. FT3, FT4 and total cholesterol were positively correlated and characterized the second axis (PC2). The third axis (PC3) was characterized by the association of triglycerides, TyG index and HDL cholesterol. TF appeared to be related to lipemic and peripheral insulin resistance profiles. A possible association between catabolic components and severity score was also noted.

**Conclusions:**

A low FT3 level is common among MIS-C. TF may be useful to define the impact of MIS-C on children’s health and help delineate long term follow-up management and prognosis.

## Introduction

Children have been found to be less affected by Coronavirus disease (COVID-19) than adults; in most pediatric cases, SARS CoV-2 is asymptomatic or only causes mild symptoms [1–4]. However, a limited number of reports describe a severe multisystem inflammatory syndrome in children (MIS-C), developing 1–2 months after acute SARS-CoV-2 infection [5–8].

According to the Centers for Disease Control and Prevention (CDC), MIS-C may occur in individuals aged < 21 years who test positive for current or recent SARS-CoV-2 infection by RT–PCR, serology, or antigen test or have been exposed to COVID-19 within 4 weeks prior to the onset of symptoms. Patients with MIS-C present with fever, laboratory evidence of inflammation and severe multisystem (≥ 2) organ involvement (cardiac, renal, respiratory, hematologic, gastrointestinal, dermatologic, or neurological) requiring hospitalization and no alternative plausible diagnoses.[9].

Several changes in thyroid hormones (TH) occur during severe acute illness and are collectively referred to as non-thyroidal illness syndrome (NTIS) [10]. NTIS has been reported in the context of infection, sepsis, trauma, burns, myocardial infarction, and malignancy [10–13]. This condition has also been described in fasting states in otherwise healthy individuals [10–13].

NTIS is characterized by a rapid decrease in serum levels of triiodothyronine (T3), hence the term ‘‘low T3 syndrome’’. In NTIS, this drop in T3 and thyroxine (T4) levels is usually not accompanied by a concomitant rise in serum thyroid-stimulating hormone (TSH). Instead, serum TSH may even decrease due to inhibition of the hypothalamic-pituitary-thyroid (HPT) axis [10, 11]. NTIS can be thought of as an adaptive response and represents the body’s attempt to decrease energy expenditure [10]. Consensus on the treatment of this condition remains undetermined and NTIS severity may be associated with poor outcomes [10]. Few studies on NTIS have been performed in the pediatric age, and often this condition remains undetected despite its association with thyroid hormone imbalances and adverse clinical outcomes.

During the COVID-19 pandemic, different studies on thyroid dysfunction and NTIS have been reported in adults [14–19]. Patients frequently develop NTIS when hospitalized in intensive care units. NTIS with its overlapping symptoms and elevated inflammatory markers represents an independent risk factor for COVID-19 severity [20]. NTIS has also been reported in mild-to-moderate COVID-19 patients; in these subjects, NTIS at admission could be useful in predicting clinical deterioration independent of viral load, age, markers of inflammation and tissue injury [20]. However, there are no detailed data on this condition in children.

We assessed thyroid function together with a series of clinical and hematological markers (metabolic, functional, inflammatory) in a cohort of children affected by MIS-C with the aim of determining a potential association between indicators of severe disease and changes in TH. We applied a multivariate analysis [21] for the characterization of mutual association patterns between markers of interest and MIS-C severity.

## Patients and methods

### Patients

We carried out a retrospective study at the Pediatric Department of the Children’s Hospital, Vittore Buzzi, Milano, from 15 November 2020 to 09 January 2021. We included all children and adolescents admitted for MIS-C according to the CDC classification.^9^ Children with pre-existing primary thyroid disease, suspicion of pre-existing hypothalamic or pituitary disease or a history of chemotherapy or radiotherapy over the last six months were excluded.

The study was conducted according to the guidelines of the Declaration of Helsinki, and approved by the Institutional Review Board of the Vittore Buzzi Hospital (Protocol Number n. 2021/ST/004). All participants or their responsible guardians were asked for and gave their written consent after being informed about the nature of the study.

### Methods

#### Clinical parameters

The patient’s physical examination included evaluation of weight, height, BMI and pubertal stage according to Marshall and Tanner as previously detailed [22].

#### Disease indicators

The following parameters were used as indicators of disease activity: complete blood count, c-reactive protein (CRP), procalcitonin, ferritin, troponin/NT-proBNP, coagulation factors, creatine kinase, electrolytes, fasting plasma glucose, triglycerides (TG), total and HDL cholesterol. We also measured insulin resistance (IR) using the triglyceride glucose (TyG) index as a simple surrogate; calculated as follows: ln[fasting triglycerides (mg/dl) × fasting plasma glucose (mg/dl)/2]) [23]. Intravenous immunoglobulin and corticosteroids were also included in the therapeutic protocol.

To define the severity of multisystemic involvement, a score was devised by our group using the parameters defined in the literature and detailed in Table 1; essentially by summing the severity scores of each organ involved.

**Table 1:** Severity score according to system involvement

Organ	Score		
	0	1	2
Kidney	Normal creatinine	Increase in creatinine < 50% [35]	Increase in creatinine > 50% [35]
Heart	Ejection fraction > 45% [36]	Ejection fraction 36–45% [36]	Ejection fraction < 36% [36]
Gastrointestinal system	None	Abdominal symptoms [37]	Acute abdomen [38]
Central nervous system	None	Neurological symptoms [37]	Impaired consciousness [39]
Lung	None	Non invasive respiratory support	Invasive ventilation
Immune (fever)	< 3 days	3–7 days [40]	> 7 days [40]
Skin/mucosal involvement	None	Skin	Skin + mucosal

#### Thyroid function

The evaluation of thyroid function included: measurement of serum free T3 (FT3), free T4 (FT4) and TSH obtained within 24 h of admission. We used the chemiluminescence immunoassay by Alinity/Abbott system (limit of detection: TSH 0.0083 µIU/ml; FT3 1.25 ng/dl; FT4 0.42 ng/dl) and the following normal ranges: FT3 3.5–6.3 pmol/L; FT4 9–19.3 pmol/L and TSH 0.5–4.2 µIU/mL.

NTIS was defined as any abnormal TF result in the presence of critical illness and absence of a pre-existing abnormality in the hypothalamic-pituitary-thyroid axis. This definition allows for the identification of usual and unusual patterns of NTIS.

## Statistical analysis

To characterize the patients and account for multi-organ involvement we created an overall severity index. This new discrete variable referred to as the “Overall Severity Score” was calculated by summing individual severity scores for each organ. Continuous variables are presented as ranges, median and quartiles with mean ± standard deviation, suitable for symmetric distributions; categorical variables are expressed as numbers and percentage of patients.

To explore the hematologic and metabolic characteristics of the patients and to obtain a comprehensive view of their distribution, a univariate statistical analysis was performed. This included a scatterplot and bar chart, showing the mean and standard deviation values. Pearson (linear) and Spearman (monotonic) correlation coefficients were computed and reported in a correlogram.

A principal component analysis (PCA) was performed to thoroughly investigate associations among the parameters since pairwise associations do not consider joint relationships with other variables. This method rotates the axes of the multivariate space of the original variables, along orthogonal directions of maximal variance (principal components PCs), and creates a new space defined by the PCs. Each of the PCs is characterized by a percentage of explained data variance. If a relevant amount of variance is explained by the first PCs, the projection of the variables as vectors in the subspace defined by the new axes is useful for exploring correlated structures present in the data. If variables are correlated with each other, the vectors are projected close together (positive correlation) or conversely with a distant alignment (negative correlation). An angle approaching 90° among the vectors indicates a lack of correlation. Moreover, if the subspace defined by the two principal components describes the variables well, their vectors are projected towards a circle of radius unity also known as the “circle of correlation”, otherwise they tend to be projected close to the origin of the axes. All of the variables were considered in the analysis and used to compute the principal components. The variable Overall Severity Score was considered a supplementary variable and so it was projected into the multivariate space defined by the PCs without participating in their composition. PCA allowed us to reduce the dimensionality of the dataset by projecting each data point into the first few principal components (up to four) while preserving as much of the data variation as possible. All of the statistical analyses were conducted with R software (version 4.0.0).

## Results

### Patient characteristics

In the study, we included 26 children and adolescents (19 M/7F) with a median age of 10.69 (5.78, 13.28) years. In Tables 2 and 3, the demographic, clinical and biochemical characteristics of the patients are reported. Normal weights were reported in all of the patients; in 16 cases (61.5%) a weight loss (average 2.50 ± 2.27 kg; 6.41 ± 4.87%) was reported before admission.

**Table 2:** Demographic and clinical characteristics of the enrolled patients

Variable	Value
Age (years)*	10.69 (5.78–13.28)
Sex (M/F)	20/6
Tanner stages (n. of patients)	
Tanner 1	13
Tanner 2–3	7
Tanner 3–4	6
BMI *	17.45 (16.24–20.43)
BMI *z* Score*	0.26 (− 0.40–1.10)
Duration of hospitalization in pediatric intensive care unit	3.0 (2.0–4.0)
Total duration of hospitalization	12.00 (10.00–15.75)
Overall severity score*	9.00 (8.00–10.00)
Severity Score 2 (n. of patients)	
Kidney	2/26
Heart	5/26
Gastrointestinal system	0/26
Central nervous system	1/26
Lung	1/26
Immune (fever)	6/26
Skin/mucosal involvement	4/26
Positive outcome	26/26

*Data are expressed as the median and 25% and 75% quantiles

**Table 3:** Biochemical characteristics of the enrolled patients

Variable	Value	Normal value	Number of patients with pathological values
Fasting blood glucose levels (mg/dl)*	111.0 (90.5–130.0)	< 100	18/26
Fasting triglycerides (mg/dl)*	195.0 (114.0–298.0)	< 150	14/25
Trygliceride glucose index*	9.221 (8.921–9.615)	< 7.88	25/25
Total cholesterol (mg/dl)*	120.0 (85.0–164.0)	< 190	4/23
HDL cholesterol (mg/dl)*	15.00 (4.00–24.00)	< 40	22/23
Leucocytes (× 10^9/L)*	9.64 (6.63–14.01)	< 2 years: 6–17; 2–8 years: 5.5–15.5; 8–16 years: 4.5–13.5; > 16 years: 4.5–13	11/26
Platelets (× 10^9/L)*	170.5 (121.5–230.0)	150–500	9/26
Hemoglobin (g/L)*	107.50 (91.25–113.00)	< 2 years > 105; 2–12 years > 115; 12–18 years males > 130; 12–18 years females > 120	24/26
Creatinine (mg/dl)*	0.5250 (0.4225–0.5777)	0.7–1.2	0/26
Urea (mg/dl)*	30.50 (19.75–45.00)	19–50	6/26
C-reactive protein (mg/dl)*	259.6 (119.9–298.7)	< 10	26/26
Procalcitonin (µg/L)*	6.55 (2.15–18.45)	< 0.5	25/26
NT-proBNP (ng/L)*	9282 (4214–14,760)	< 125	26/26
Troponin (ng/L)	39.50 (16.75–112.00)	< 15	21/26
Fibrinogen (g/L)*	6.430 (6.430–6.780)	< 4	26/26
INR*	1.350 (1.240–1.427)	0.84–1.16	23/26
D-dimer (µg/L)*	2734 (2003–4154)	< 200	26/26
Creatine kinase (U/L)*	63.50 (35.25–130.25)	Males: 47–322 Females: 29–201	3/26
Na (mEq/L)*	131.0 (128.0–134.0)	135–145	19/26
K (mEq/L)*	3.500 (3.000–3.900)	3.5–5	13/26
Ferritin (µg/L)*	675.5 (402.0–1491.8)	7–140	26/26
FT3 (pmol/L)	2.45 (1.92–3.15)	3.5–6.3	22/26
FT4 (pmol/L)	11.65 (10.32–13.73)	9–19.3	7/26
TSH (µIU/mL)	2.16 (1.18–3.00)	0.5–4.2	3/26

In particular, the parameters considered to define the MIS-C diagnosis are reported. Additionally, the triglyceride glucose (TyG) index is reported as a surrogate of insulin resistance

*Data are expressed as the median and 25% and 75% quantiles

Oxygen therapy was administered in 21/26 subjects. Twenty patients were hospitalized in the pediatric intensive care unit (PICU); the median PICU stay was three days (2, 4). Ventilator support was required in 11 cases, including continuous positive airway pressure in 10 patients and high flow nasal cannula in one case (median days of ventilation, 3 (2.0–4.5). Overall, a median hospitalization duration of 12.50 (10.00–16.75) days was recorded. Outcome was positive, all patients making a full clinical recovery.

### Changes in thyroid hormones

Biochemical data on the study patients are reported in Table 3. 88.4% (23/26) of the patients exhibited NTIS. Median hormone levels were FT3 2.45 pmol/L (1.92, 3.15), FT4 11.65 pmol/L (9–19.3) and TSH 2.16 µIU/mL (1.18, 3.00). In Table 4, the distribution and patterns of FT3, FT4 and TSH values are reported.

**Table 4:** Distribution and patterns of FT3, FT4 and TSH values

Parameter	Number of patients (%)
Hormonal levels in all patients (*n* = 26)	
FT3 (nv 3.5–6.3 pmol/L)	
Normal	4 (15.4)
Low	22 (84.6)
FT4 (nv 9–19.3 pmol/L)	
Normal	19 (73.1)
Low	7 (26.9)
TSH (nv 0.5–4.2 µIU/mL)	
Normal	23 (88.5)
Low	2 (7.7)
High	1 (3.8)
Hormonal pattern in patients with NTIS (*n* = 23)	
Low FT3 only, *n* (%)	15 (65.3)
Low FT3, Low FT4, *n* (%)	5 (21.7)
Low FT3, Low FT4, Low TSH	2 (8.7)
High TSH, *n* (%)	1 (4.3)

*nv* normal values, *NTIS* non-thyroidal illness syndrome

Some subjects with NTIS had abnormal levels of only one hormone (15/23, 65.2%), while others had variable combinations of hormone abnormalities (8/23, 34.7%). In particular, we noted: an isolated decrease in the FT3 level in 15/23 (65.3%) subjects; low FT3 and FT4 levels in 5/23 (21.7%); low FT3, FT4 and TSH values in 2/23 cases (8.7%) and an increase in TSH level in one patient (4.3%).

### NTIS and multivariate analysis

**Fig. 1:**
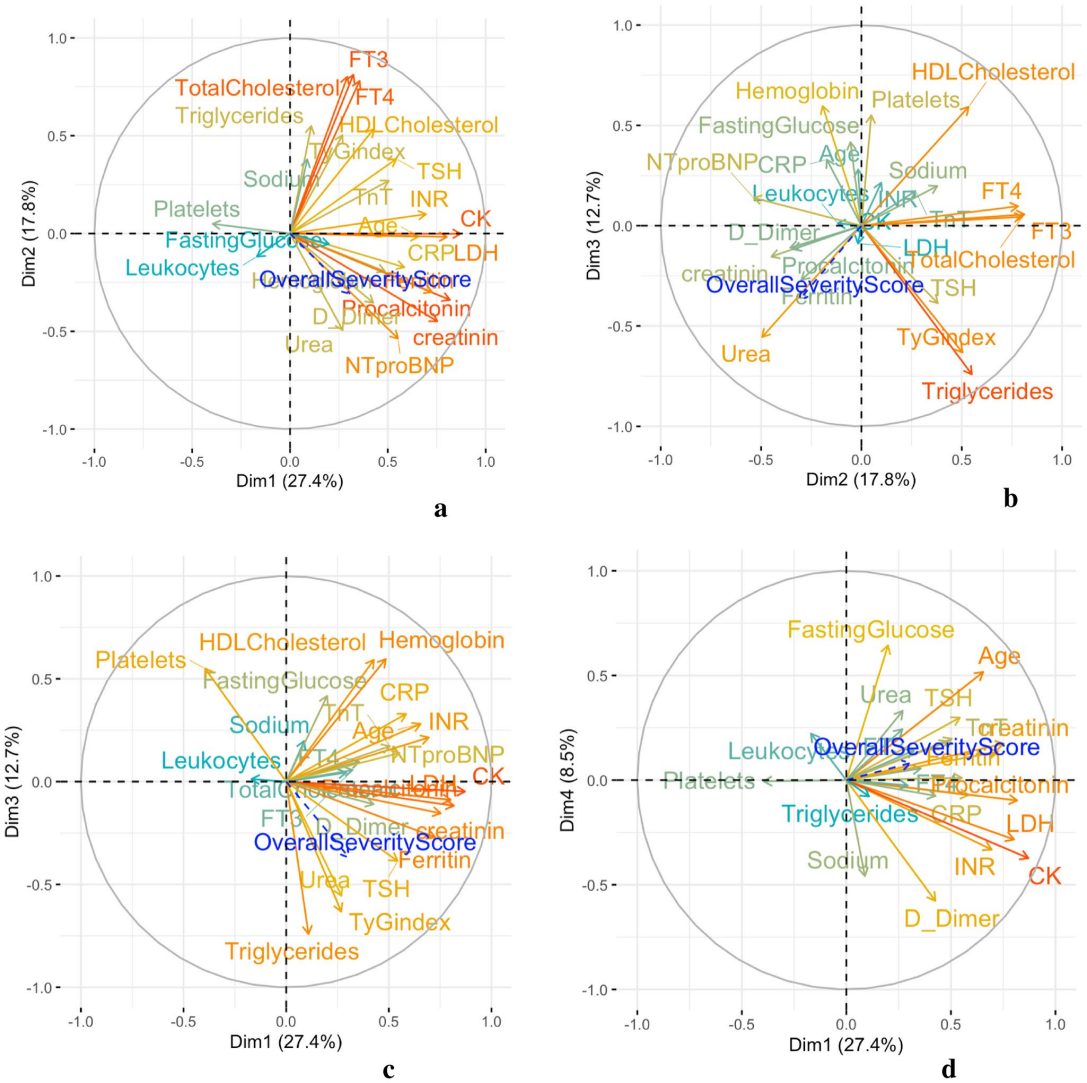
The panel shows the four subspaces defined by the first four PCs (i.e., Dim1, Dim2, Dim3, Dim4). When the subspace defined by the two principal components describes the variables well, these are projected towards a circle unity radius, also known as the circle of correlation. The warmer the color of the vector, the better that variable is described by the plane defined by the PCs; the cooler the color of a variable vector, that variable is not as well described by the plane. As can be seen in (**a**, **b**), variables FT3, FT4 and the metabolic variable total cholesterol are well represented in the plane defined by Dim1 and Dim2 and in the plane defined by Dim2 and Dim3. Indeed, these are all positively correlated to each other and positively correlated to the second dimension. Moreover, in (**a**, **c**, **d**), the variables related to organ damage and inflammation are well represented in the planes and positively correlated to each other and to the first dimension (Dim1). Finally, in (**b**, **c**) where Dim3 participates in the definition of the plane, the variables TyG index and triglycerides are, as expected, strongly correlated to each other, and associated with the third dimension. In all of the planes (**a**, **b**, **c**, **d**) the variable Overall Severity Score is projected passively and, as expected, its vector points in the same direction as the variables related to organ damage and inflammation

**Fig. 2:**
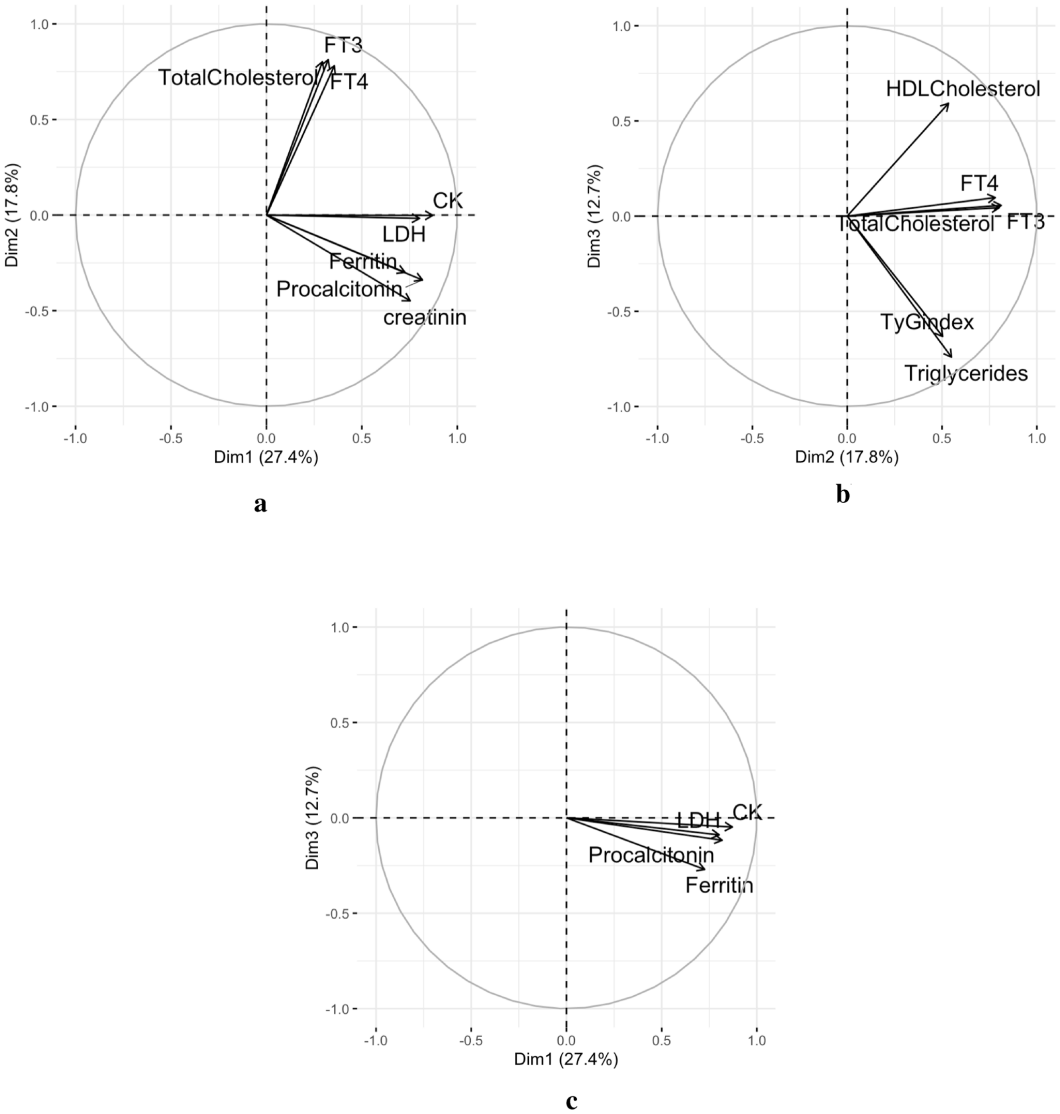
The panel shows the four subspaces defined by the four PCs (i.e., Dim1, Dim2, Dim3 and Dim4). In this case, cut-off values were applied to show only the variables that were well represented by the planes defined by the PCs. As shown, the circles of correlation confirm the results displayed in Fig. 1, in which variables FT3, FT4 and total cholesterol are dependent on the presence of Dim2, those related to organ damage and inflammation are dependent on the presence Dim1 and the variables TyG index and triglycerides are dependent on the Dim3

PCA analysis does not allow for any missing values in the matrix. For this reason, only 20 observations from the 26 patients were considered in the descriptive and correlation analysis, since they displayed finite values for all the variables considered. In the bi-plots displayed in Figs. 1 and 2, some variables are strongly associated and form clusters.

The multivariate PCA analysis of the dataset showed that the first six principal components explained 80% of the variation in the data (PC1 = 27%, PC2 = 18%, PC3 = 12%, PC4 = 8%, PC5 = 7%, PC6 = 6%). We focused our attention on the first three dimensions since they explain much greater variation in the data with respect to the other three and are well characterized by specific variables of interest. While the variables related to organ damage and inflammation such as CK, LDH, procalcitonin and creatinine are represented in the first dimension (PC1), FT3, FT4, total cholesterol were all grouped together, and they characterize the second axis (PC2). Moreover, the third axis (PC3) was mainly characterized by a group of variables related to metabolic features (triglycerides, TyG index and HDL cholesterol).

**Fig. 3:**
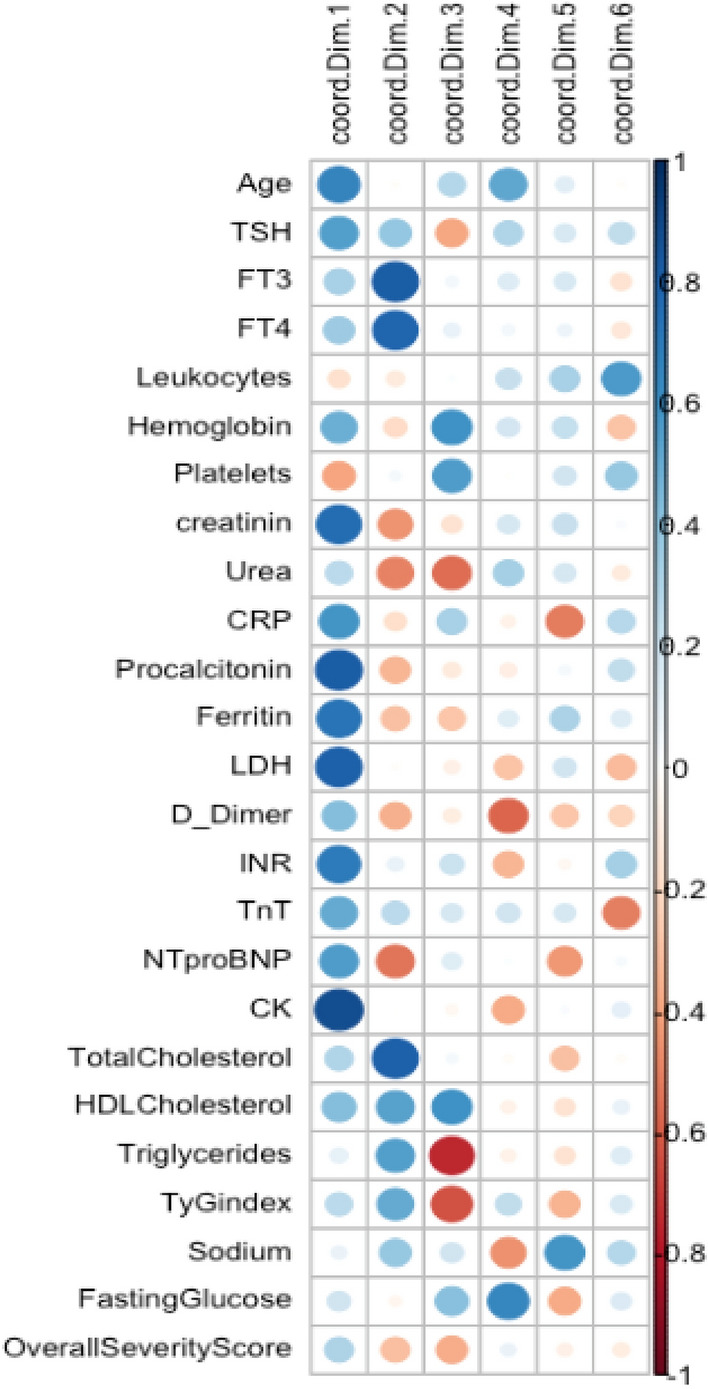
The panel shows a correlogram in which the correlations observed between the variables and the dimensions in the PCA are displayed. Correlations are indicated with color and dimension-coded circles. The greater the intensity of the color and the larger dimension of the circle, the greater the correlation; and vice versa. The blue color spectrum is associated with a positive correlation, while the red color spectrum is associated with a negative correlation. The first dimension is strongly correlated with the variables related to organ damage and inflammation and also with the Overall Severity Score. Whereas, the second dimension is strongly correlated with variables FT3, FT4 and total cholesterol; positively correlated with the metabolic variables; and negatively correlated with the Overall Severity Score. It is notable that the third dimension—strongly and negatively associated with the variables TyG index and Triglycerides—is the most negatively associated with the Overall Severity Score

Considering the graphs showing the projection of variables as vectors in the new plane defined by the PCs (Fig. 1), it was clear that in the plane defined by the first two PCs, variables FT3, FT4, and total cholesterol all grouped together and strongly correlated to each other. In the same subspace, the variables related to organ damage and inflammation (CK, LDH, procalcitonin, creatinine) were positively correlated with each other. As expected, by passively projecting the newly generated variable coding for the Overall Severity Score, this variable was projected in the same direction as the vectors of the variables related to organ damage and as such positively correlated with these. It was also evident that a slight negative correlation existed between the group of variables FT3, FT4, total cholesterol and the Overall Severity Score. To better visualize only the variables that were robustly described by the planes defined by the pairwise consideration of the PCs, we produced other graphs by considering cut-off values (cos2 = 0.60) for the contribution of the variables in the plane. As expected, the variables mentioned above were all present in the graphs of the planes defined by PC1, PC2 and PC3, Fig. 2. These considerations were confirmed by a comparison in the correlogram showing the pairwise correlations between the variables and each dimension resulting from the PCA (Fig. 3).

## Discussion

We observed that more than 90% of children with Sars-Cov2 associated MIS-C exhibited NTIS. An isolated decrease in the level of FT3 and a FT3 decrease associated with an increase in TSH level were the most and least represented patterns, respectively. NTIS detection, based on the evaluation of FT3, FT4 and TSH levels, may be helpful in the management of children with MIS-C requiring hospitalization and this phenomenon may be considered an adaptive mechanism to preserve energy during critical illness.

Acute and chronic Illnesses may induce profound modulations and interactions in a number of neuroendocrine systems including changes within the HPT axis [24–26]. Data in the literature suggest that SARS-CoV-2 may also have an impact on thyroid tissue and function [16, 25, 27]. Different mechanisms have been proposed to explain the underlying pathogenic mechanism [25]. The first suggests that the high ACE2 and TMPRSS2 expression in the thyroid may facilitate Sars-Cov2 entry. A second potential explanation is systemic immune activation in response to SARS-CoV-2 infection may cause thyroid damage. A third hypothesis is a selective transient pituitary dysregulation, due to both the direct cytotoxic effect of the virus at the pituitary level and an indirect effect via the activation of proinflammatory cytokines, which produce a “cytokine storm” that would, in turn, induce NTIS [24–26, 28–30].

NTIS is characterized by TH abnormalities that represent imbalances in hormonal production, metabolism, and action [32]. Usually, TH abnormalities are observed in two distinct temporal phases. In the first phase, acute modifications in peripheral metabolism of TH are predominant. At this stage, low T3 syndrome occurs and can be explained by changes in TH binding, TH peripheral uptake and type-1 deiodinase and type-3 deiodinase expression and activity [31]. In the second phase, disturbances of a neuroendocrine origin predominate [31].

In this study we report on NTIS among SARS-CoV-2 associated MIS-C. We observed a high prevalence of low T3 syndrome, which is considered a useful adaptation to preserve energy during critical illness [24–26, 28–30]. As in our children, patients with critical illness experience a concomitant weight loss due to a hypercatabolic state [33]. In this situation, decreased TH availability reduces energy expenditure inducing a beneficial effect [32]. Additionally, increased D3 activity may optimize the bacterial killing capacity of neutrophilic granulocytes [33]. Even though the first phase of TH abnormalities was predominant in our population, the presence of different patterns of FT3, FT4 and TSH abnormalities in our NTIS patients, suggests that cytokines could also be implicated in suppression of the hypothalamic-pituitary axis [25, 26, 28].

In different cohorts of patients, such as subjects with community-acquired pneumonia, low T3 syndrome has been identified as an independent risk factor associated with a poor 30-day mortality rate [33]. This effect may be attributed to large amounts of inflammatory cytokines, including tumor necrosis factor (TNF)-α, interleukin (IL)-1, IL-6, and inversely with TH levels, which are present during critical illness [34].

Positive outcomes were achieved in all of our patients. The multivariate pattern analysis showed that variables related to organ damage, inflammation and severity of multisystemic involvement did not correlate with thyroid dysfunction. Additionally, other hematological parameters, such as hemoglobin, platelets, leukocytes were not associated with TF??. These results suggest that low T3 syndrome may be a useful adaptation to preserve energy during long periods of critical illness rather than during acute episodes [35]. However, data from the literature evidence that abnormalities in TH metabolism during systemic illness are variable in different clinical settings; thus the role of organ involvement in TH changes cannot be excluded.

Although MIS-C varies in severity, the majority of our patients were seriously ill. Our multivariate analysis showed that TF was related to lipemic profiles, particularly triglycerides and HDL cholesterol, and the TyG index of peripheral insulin resistance, suggesting that a hypercatabolic syndrome exists. Future studies on the correlation between catabolic components and overall severity score should confirm these data.

This analysis was further complicated by the fact that NTIS pathogenesis is complex and multifactorial. Even though, the optimal therapeutic approach in children with MIS-C has not been defined, the majority of patients are treated with standard therapeutic protocols including: glucocorticoids, intravenous immunoglobulin and anticoagulant agents. Glucocorticoids are known to affect serum TSH levels in humans and heparin is known to interfere with free thyroid hormone assay [25]. However, due to the urgency of patient’s care, TH evaluation is generally performed at admission, prior to the start of therapy. Therefore, a hypothetical iatrogenic mechanism may be reasonably excluded. Although, an evaluation of thyroid autoantibodies was not performed to exclude an autoimmune thyroid disorder, all of the enrolled patients had no previous known thyroid disease and no anamnestic medical record influencing thyroid function. Additionally, the young age of the patients and the male predominance of NTIS supports a non-autoimmune pathogenesis.

We recognize that the small sample size limits the strength of the analysis, thus we are working to increase the sample size to extend and validate these first results particularly in relation to the Disease Severity Score. However, we felt these preliminary results were interesting as they explore the mutual association between metabolic and hormonal patterns and their impact on the severity of the clinical condition.

In conclusion, NTIS is common among Sars-Cov2 associated MIS-C subjects. NTIS may be interpreted as evidence of an adaptation to preserve energy during critical illness and hypercatabolism. Since the endocrine system critically coordinates and regulates major metabolic and biochemical pathways, an endocrinological profile may prove useful in the diagnosis of MIS-C and help define its real its real impact on children's health, and assist in developing the best long term follow-up management plan and prognosis.

## Data Availability

Data are available in the article or supplementary material.
